# Replicative Study in Performance-Related Genes of Brazilian Elite Soccer Players Highlights Genetic Differences from African Ancestry and Similarities between Professional and U20 Youth Athletes

**DOI:** 10.3390/genes14071446

**Published:** 2023-07-14

**Authors:** Tane Kanope, Caleb G. M. Santos, Feliciana Marinho, Gustavo Monnerat, Mario Campos-Junior, Ana Carolina P. da Fonseca, Verônica M. Zembrzuski, Miller de Assis, Michael W. Pfaffl, Eduardo Pimenta

**Affiliations:** 1 UFMG Soccer Science Center, School of Physical Education, Physiotherapy and Occupational Therapy, Federal University of Minas Gerais, Belo Horizonte 31250810, Brazil; tane.ufrj@gmail.com (T.K.); empimenta@uol.com.br (E.P.); 2 Department of Animal Physiology and Immunology, School of Life Sciences, Technical University of Munich, Weihenstephaner Berg 3, 85354 Freising, Germany; 3 Teaching and Research Division, Brazilian Army Institute of Biology, Rio de Janeiro 20911270, Brazil; 4 Instituto Hermes Pardini SA, Vespasiano 33200000, Brazil; 5 Instituto de Biofisica Carlos Chagas Filho, Universidade Federal do Rio de Janeiro, Rio de Janeiro 21941902, Brazil; 6 Human Genetics Laboratory, Oswaldo Cruz Institute, Oswaldo Cruz Foundation (Fiocruz), Rio de Janeiro 21040360, Brazil; 7 Laboratory of Immunopharmacology, Oswaldo Cruz Institute, Rio de Janeiro 21045900, Brazil

**Keywords:** population genetics, genetic distance matrix, SNP panel, soccer, athletic performance

## Abstract

Classically, genetic association studies have attempted to assess genetic polymorphisms related to human physiology and physical performance. However, the heterogeneity of some findings drives the research to replicate, validate, and confirmation as essential aspects for ensuring their applicability in sports sciences. Genetic distance matrix and molecular variance analyses may offer an alternative approach to comparing athletes’ genomes with those from public databases. Thus, we performed a complete sequencing of 44 genomes from male Brazilian first-division soccer players under 20 years of age (U20_BFDSC). The performance-related SNP genotypes were obtained from players and from the “1000 Genomes” database (European, African, American, East Asian, and South Asian). Surprisingly, U20_BFDSC performance-related genotypes had significantly larger F_ST_ levels (*p* < 0.00001) than African populations, although studies using ancestry markers have shown an important similarity between Brazilian and African populations (12–24%). U20_BFDSC were genetically similar to professional athletes, showing the intense genetic selection pressure likely to occur before this maturation stage. Our study highlighted that performance-related genes might undergo selective pressure due to physical performance and environmental, cognitive, and sociocultural factors. This replicative study suggests that molecular variance and Wright’s statistics can yield novel conclusions in exercise science.

## 1. Introduction

Physical performance is a multifactorial trait to which genetic data can potentially be applied for precision medicine approaches [1]. Classically, genetic association studies (GASs) are used to better understand the effects of genetic variability. Hundreds of previous studies have attempted to assess genetic polymorphisms and link their allele frequencies to human physiology and performance phenotypes. Based on GAS polymorphisms, there are lists of genes commonly associated with sports performance (Table 1) which support additional deeper investigations using animal models, human tissues, and cells to validate the candidate genes.

Thus, important single-nucleotide polymorphisms (SNPs), like the non-sense rs17602729 in the *ACTN3* gene, have had their mechanisms described and related to sports performance. In this case, the absence of α-actinin-3 releases more calcineurin favoring endurance adaptation [2]. Additionally, the total or partial lack of adenosine monophosphate deaminase-1 due to *AMPD1* rs1760272, a key enzyme for purine metabolism, was previously related to ATP recovery and fatigue during exercise, a core valence for soccer players. In addition, peroxisome proliferator-activated receptor (PPAR)-related genes and their SNPs likely exert a complex, modulatory influence on fatty acid and carbohydrate oxidation pathways. However, their specific mechanisms remain to be understood [3]. Moreover, SNPs like *FTO* rs9939609 appeared in more than one GAS related to fitness and body fat but without real validation [4].

Alleles that enhance essential traits for specific physical activities likely experience selective pressures in elite athletes without consistently affecting unrelated genes [5]. However, the heterogeneity of some findings, even at the beginning of the genomic era, drives the research for replication, validation, and confirmation of the findings as essential aspects for ensuring the quality and applicability of their results in sports or medical sciences. Replicating positive findings favors the validity of genotype–phenotype associations and will avoid biases. For this reason, it is essential to observe various aspects using established analysis methods, genetic variants, the definition of phenotype, and ethnic/admixed groups, as well as independent but similar data sets [6].

**Table 1:** Summarized information about single-nucleotide polymorphism (SNP) genetic panel.

Groups of SNPs	Previous Reports
*ACTN3* rs17602729	Most studied polymorphism for sports science related to strength/power performance [2,7]
*AMPD1* rs1760272	Nonsense; related to ATP recovery and fatigue [8]
*PPARGC1A, MCT1, CYP1A2* rs8192678G, rs10494, rs76255134	Metabolic pathways and energy expenditure [9,10]
*COL5A1, MMP3* rs12722, rs591058	Stability and health of muscle structure [11,12]
*FTO* rs9939609	Physical fitness in several whole-genome GASs [13]
*I6 CHRM2, I5 CHRM2* rs8191992, rs324640	Heart rate recovery and electrical conduction [14]

*ACTN3* (α-actinin-3), *AMPD1* (adenosine monophosphate deaminase 1), *PPARGC1A* (PPARG coactivator 1 alpha), *MCT1* (solute carrier family 16 member 1), *CYP1A2* (cytochrome P450 family 1 subfamily A member 2), *COL5A1* (collagen type V alpha 1 chain), *MMP3* (matrix metallopeptidase 3), *FTO* (α-ketoglutarate-dependent dioxygenase), *I6 CHRM2* (cholinergic receptor muscarinic 2—intron 6), *I5 CHRM2* (cholinergic receptor muscarinic 2—intron 5).

The recent results in 25 male Brazilian soccer players [15] showed an alternative use of genetic panels and GASs, approaching a concurrent contribution of SNPs based on genetic matrix distance and ethnic aspects between athletes or public genomic databases, like the “1000 Genomes” database. Notably, matrix distance-based studies using proteomic and metabolomic data in a pairwise or multidimensional analysis have shown biomarker patterns by clustering groups based on moments of acute exercise [16,17,18], performance [19,20,21], and sports disciplines or physiological variables [22]. However, as a population genetics method, human research on this subject has been mainly applied to the forensic field. Thus, this short communication aims to investigate the replicability of a “genetic distance approach” based on SNPs and Wright statistics for genetic distances [23], as an alternative to traditional GASs, in an unrelated, new, younger, and larger population of soccer athletes, to determine if the U20 athletes are genetically related to professional ones and other ethnicities available in 1000 Genomes database.

## 2. Materials and Methods

We selected an independent study group of soccer players for replication. We performed a complete sequencing of 44 genomes from male Brazilian first-division soccer players under 20 years of age (U20_BFDSC) to compare them with a group of 25 probands (BFDSC). DNA was obtained from buccal cells using a MagMAX™ DNA Multi-Sample kit (ThermoFisher, Waltham, MA, USA). Genomic libraries were constructed from 1 μg of DNA using a TruSeq DNA PCR Free kit (Illumina^®^, San Diego, CA, USA). The libraries were sequenced on the NovaSeq6000 platform (Illumina^®^, San Diego, CA, USA) with an average depth of 44×. Read mapping and genome assembly were performed based on the GRCh38 version of the human genome using DRAGEN Germline App v3.7.5 available in the BaseSpace cloud file deposit (Illumina, USA). Variant calling was performed using the DRAGEN tool™. The SNP chosen genotypes were obtained from VCF files and checked manually from BAM files using the Integrative Genomics Viewer (IGV) tool (Broad Institute, University of California, USA) [24]. Hardy–Weinberg equilibrium was tested using the patch “genetics” of R software v3.02 (Vienna, Austria).

Genotypes from European, African, American, East Asian, and South Asian populations were obtained from the 1000 Genomes database using the Ensembl home browser (www.ensembl.org, accessed on 1 October 2022). The genetic distances based on molecular variance (F_ST_) were calculated using Arlequin software v3.5 (Bern, Switzerland) [25]. The principal component analysis (PCA) was performed using Past3 software (Oslo, Norway), and the phylogenetic tree was constructed using Interactive Tree Of Life (iTOL) v6 [26] (available at https://itol.embl.de, accessed on 21 January 2023). This study was approved by the Local Research Ethical Committee (76189817.0.0000.5235 and 69253417.1.0000.5149) according to the ethical standards of the Helsinki Declaration. All participants signed a written consent form to participate.

## 3. Results

Following the genetic distance matrix based on population genotypes [27], we analyzed an additional larger cohort of Brazilian first-division soccer players under 20 years of age (n = 44, U20_BFDSC) to compare the new genetic data with another cohort of professional players (n = 25, BFDSC) and 2503 genomes from the 1000 Genomes database (European = 502, African = 661, American = 347, East Asian = 504, South Asian = 489). Unlike traditional genetic association studies that study SNPs individually, we used a sports performance-related SNP panel in a genetic distance approach based on Wright’s statistics [23].

After testing for replicability, our results corroborated the previous minor cohort of 25 participants [1]. Although ancestry studies based on genome-wide arrays have shown an important similarity between south-eastern Brazilian and European, African, and Native American populations, the panel of 10 specific performance-related SNPs used in this study revealed very low F_ST_ values between U20_BFDSC and BFDSC, European, and American populations (Table 2). Larger F_ST_ and highly significant values were observed when distance matrices based on the U20_BFDSC genotypes were compared with those of East or South Asian and African populations (*p* < 0.00001) (Table 2). F_ST_ values between 0.05 and 0.15 were classified as moderate, and above 0.15 as high genetic distance.

**Figure 1:**
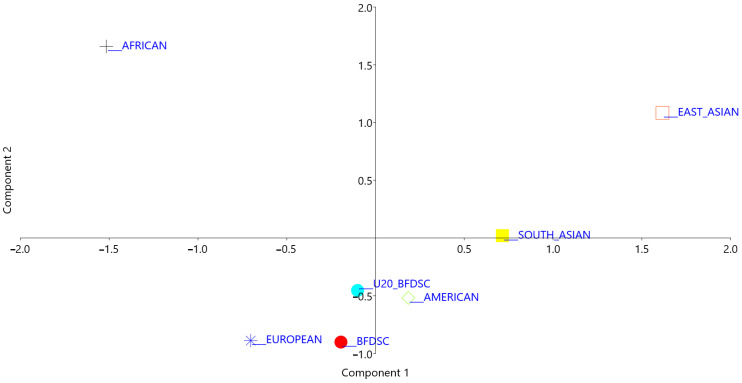
Principal component analysis regarding the distance matrix of molecular variance (F_ST_) values of continental populations from the 1000 Genomes database compared with U20_BFDSC and BFDSC.

In the multidimensional PCA analysis, the U20_BFDSC group clustered closely and in the same quadrant as the European and the previous BFDSC group population, near and in different quadrants from the American population, but far from the African or East Asian populations (Figure 1). The PCA was performed using the eigenvalue strategy for variance analysis. The first (58.4%) and the second (32.5%) components accounted for around 91% of the total variance, confirming the significance of the F_ST_ values and the statistical robustness of the model.

No statistically significant genetic molecular variances (F_ST_) were observed between the U20_BFDSC and BFDSC groups, and they remained very close on the phylogenetic tree (Figure S1), supporting replication of the results. In addition, we cannot ignore the possibility that linkage disequilibrium of non-genotyped SNPs may contribute to the clustering results obtained through the ten chosen markers.

## 4. Discussion

GASs can provide valuable insights, but achieving statistical confidence requires large sample sizes. To evaluate GAS results, a significance level of 5 × 10^−8^ based on classical Bonferroni’s correction is typically used [28]. However, population genetics studies using Wright’s statistics demonstrated significant group clustering with lower sampling. For example, in non-human models, matrices of genetic distances based on genotypes could separate genetically close groups containing as many as 17, 26, or fewer individuals by employing appropriate sets of SNPs [27,28]. Theoretical studies based on simulations indicate that even small sample sizes can produce accurate and unbiased estimates of F_ST_ when using enough and related bi-allelic markers like SNPs [29].

The Brazilian population is highly diverse and admixed, with a significant ancestral influence from Caucasians (61–77%), Africans (12–24%), Native Americans (10–15%), and Asians (10%) [30,31]. Surprisingly, previous cohorts [15] have shown lower African-related ancestry in sports-related genes, prompting a replicative investigation in a larger, independent cohort. Additionally, recent findings based on total genotype scores described that the genetic profile of elite youth soccer players depends on their maturity status, based on a comparison of ages around 11 and 17 years old [32,33]. Although Wright’s statistics can distinguish slight genetic differences, the U20_BFDSC group was not different from the BFDSC group. It is reasonable to hypothesize that the U20 category may resemble professional athletes, as most of the intense genetic selection pressure likely occurs before this maturation stage.

The clustering ability of molecular variance may be used even in populations with different physical phenotypes to evaluate performance. Although Wright’s statistics-based studies have a powerful tool for clustering, they are sometimes less informative because they show the global molecular variance and do not point to which specific gene plays a vital role in that phenotype. It is possible to cluster genetically related subjects or distinguish them from others, but further studies are necessary to validate a potential variant related to the phenotype. In this case, good science is mandatory in selecting homogeneous populations to identify the genetic trait. Furthermore, clustering related subjects from the same ethnicity and similar phenotypes in a multifactorial trait could be harder to distinguish and may demand more genes and DNA sequencing costs.

The study of performance-related variants could allow for the design of a supposedly optimal profile. However, selecting an athlete based on molecular variance closer to some overrepresented clusters raises ethical concerns. As a multifactorial trait, soccer performance depends on genetics and environmental aspects. Without significant validation, there is no evidence to support a genetics-based selection of subjects, which poses a real risk of data misinterpretation that could compromise the integrity of the sport.

## 5. Conclusions

Finally, our replication results highlighted the possibility that, in male elite soccer players, performance-related genes might undergo selective pressure due to physical performance and environmental, cognitive, and sociocultural factors, justifying the slightly different results concerning the African or Asian population in studies with markers of ancestry that are not related to sports performance. In addition, the U20 athletes may resemble professional athletes, although even younger players could show genetic differences between them. The possibility of using Wright’s statistics in larger samples, based on variability data from athletes’ whole genomes (consortia), could generate conclusions never seen before in exercise science.

## Figures and Tables

**Table 2:** F_ST_ values for comparisons between populations from 1000 Genomes database and U20_BFDSC.

Groups Compared to U20_BFDSC	F_ST_
BFDSC	0.00100 n.s.
AFRICAN	0.10125 ***
AMERICAN	0.03097 ***
EAST_ASIAN	0.13803 ***
EUROPEAN	0.01534 ***
SOUTH_ASIAN	0.07152 ***

n.s. not significant; *** *p* < 0.00001. Abbreviations: BFDSC = Brazilian professional first-division soccer club, U20_BFDSC = Brazilian first-division soccer club players under 20 years of age, F_ST_ = genetic molecular variances. The molecular genetic variance between populations (F_ST_ < 0.05—low, F_ST_ = 0.05–0.15—moderate, F_ST_ > 0.15—high).

## Data Availability

The datasets are fully available on request.

## Supplementary Materials

The following supporting information can be downloaded at: https://www.mdpi.com/article/10.3390/genes14071446/s1, Figure S1. Phylogenetic tree (UPGMA model) regarding genetic distance distribution from genotypes of the single nucleotide polymorphisms selected from U20_BFDSC, BFDSC, and continental populations from the “1000 Genomes database”. U20 indicates under 20 years; BFDSC indicates Brazilian first-division soccer club.
