# Supplementary material for: Replicative Study in Performance-Related Genes of Brazilian Elite Soccer Players Highlights Genetic Differences from African Ancestry and Similarities between Professional and U20 Youth Athletes
Source: Genes (Basel). 2023 Jul 14;14(7):1446. doi: 10.3390/genes14071446 (PMC10379729; doi:10.3390/genes14071446)
Supplement: Supplementary file 1 [file genes-14-01446-s001.zip › genes-2480873-supplementary.pdf]

genes

Supplemental Material

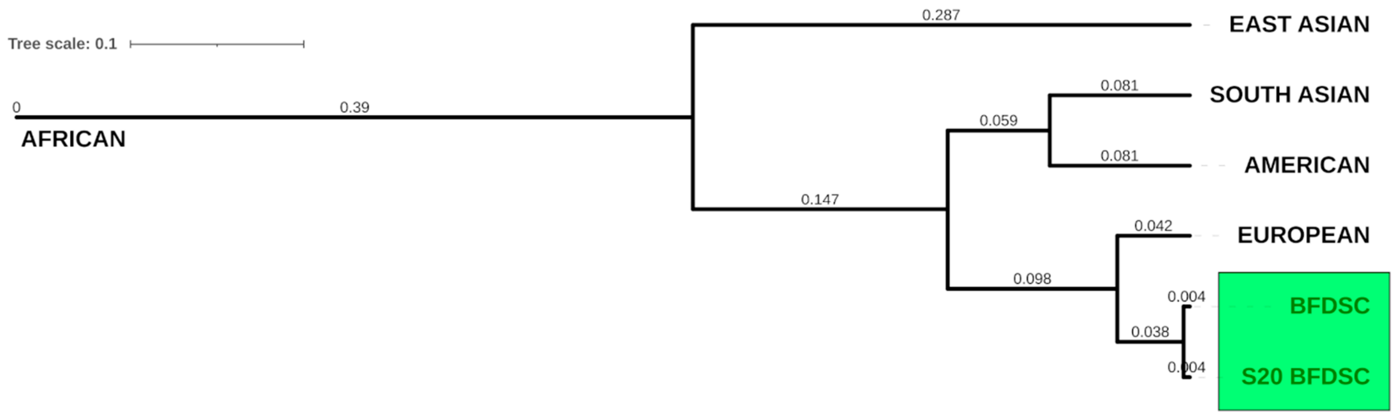

Figure S1. Phylogenetic tree (UPGMA model) regarding genetic distance distribution from genotypes of the single nucleotide polymorphisms selected from U20_BFDSC, BFDSC, and continental populations from the "1000 Genomes database". U20 indicates under 20 years; BFDSC indicates Brazilian first-division soccer club.
